# Unlocking the potential of MICROCRYSTAL ELECTRON DIFFRACTION

**DOI:** 10.1063/pt.3.5019

**Published:** 2022-06

**Authors:** Mike Martynowycz, Tamir Gonen

**Affiliations:** department of biological chemistry at UCLA

## Abstract

Atoms stick together in different ways to make the molecules that compose everything we touch and see. Our bodies are made of cells. Cells, in turn, are made of lipids, proteins, nucleic acids, metabolites, and water. Every one of those molecules is made from the same handful of atoms. But although the components are the same, the molecules differ in how many atoms they have and how those atoms are arranged in space.

Proteins are tiny biological machines. They do work at the nanoscale by moving molecules around, forming or breaking bonds, and catalyzing reactions. Structural biologists strive to determine where all the atoms reside inside proteins. The most common method uses high-energy x rays for the job. Purified proteins grow into three-dimensional crystals that act as diffraction gratings when exposed to coherent radiation. Rotating the crystal in the x-ray beam produces diffraction spots that identify the atoms’ locations inside the crystal.

But growing proteins into crystals large enough for x-ray diffraction is challenging. Indeed, the most important proteins for human health rarely grow into crystals large enough for x-ray diffraction experiments to work on them, or they are too sensitive to the radiation and break down before the data can be collected. Fortunately, a cryogenic electron microscopy (cryo-EM) method, known as microcrystal electron diffraction (MicroED),^1^ can determine protein structures from crystals as small as one billionth the size of those used in traditional x-ray crystallography.

The method uses the same cryogenic electron microscopes that biologists rely on to image macromolecular complexes or to discern the 3D structure of entire cells—techniques known as single-particle imaging and tomography, respectively. MicroED promises to open structural biology to new classes of protein nanocrystals and glean novel details from the tiny proteins.

## The structure–function relationship

Understanding what something does is powerful. It lets people know, for instance, how to fix things that are broken. Scientists refer to that understanding as the structure–function relationship. Structural biologists care about how the machinery in our bodies works and investigate how proteins operate by determining their atomic structure. Beyond many other critical functions, proteins can move sugar into cells, carry oxygen from lungs to muscles, and produce electrical signals in our brains.

**FIGURE 1.**
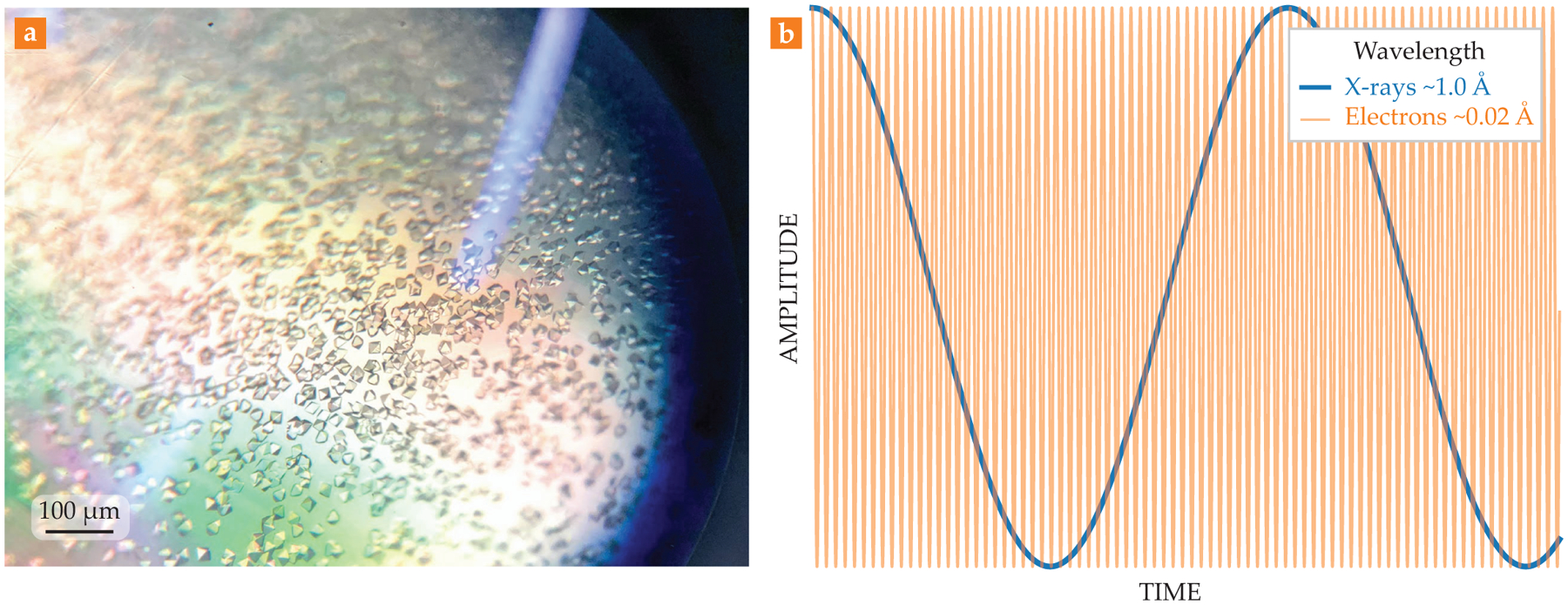
**CRYSTALS** and their diffraction. **(a)** Protein crystals of proteinase K, a serine protease, are seen through a light microscope. **(b)** The graph shows a comparison between the wavelengths of x rays (blue) and electrons (orange) typically used in diffraction experiments. With their much shorter wavelength, electrons can resolve much finer details of a biomaterial.

The first step to determining a target protein’s structure has been to grow crystals of it. Fortunately, many proteins can arrange into a repeating 3D pattern to make crystals. Such crystals are grown by isolating the pure protein and mixing it with various salts and additives that coerce the protein into small, ordered clumps that then grow outwardly into beautiful, faceted shapes, as shown in figure 1a. Those crystals are then interrogated by a beam of x rays.

At large synchrotron light sources, strong magnetic fields whip electrons around circular tracks at relativistic speeds. The accelerating electrons emit a broad spectrum of light. Such light sources are enormous, with circumferences typically on the scale of hundreds of meters. Stretching out from those rings are end stations, at which the electromagnetic spectrum is filtered and an emerging x-ray beam is used for experiments.

Protein crystals placed in the path of those beams diffract a small fraction of the x rays into detectors that record their pictures—tiny spots known as reflections, similar to the ones shown in the opening image. Calculations based on both the locations and intensities of the reflections build up a map of the positions of every atom inside the protein.

Although growing crystals is standard practice for x-ray diffraction, growing protein crystals large enough to be studied can take years or fail altogether. That bottleneck has led many structural biologists to search for other methods to determine a protein’s structure.

## Cryo-EM in retrospect

The 2017 Nobel Prize in Chemistry was awarded to Jacques Dubochet, Joachim Frank, and Richard Henderson for their development of cryo-EM of biomolecules in solution (see Physics Today, December 2017, page 22). Traditional light microscopes magnify small objects by focusing light through glass lenses—an achievement limited by the wavelength of visible light. Electrons, by contrast, have a wavelength far lower than visible light—smaller even than typical x rays (see figure 1b). And because they carry both charge and mass, electrons can be accelerated to high velocity using electromagnetic lenses. The upshot: Electron microscopes produce images with details that are far finer than can be seen with a light microscope.

Even so, imaging biological material as small as an individual protein is difficult. High-energy electrons must propagate in a vacuum, which is incompatible with a liquid environment—the natural home for most proteins. And those electrons can damage biological materials. To circumvent those problems, researchers developed methods to freeze the sample quickly enough that the protein’s liquid surroundings cannot crystallize. They leave the proteins embedded in a thin layer of vitrified, amorphous ice. The frozen, hydrated state exists at a liquid-nitrogen temperature of about −320 °F, an environment that *is* compatible with electron microscopy.

Early cryo-EM studies that preceded the development of rapid-freezing techniques typically focused on proteins that grew into large, 2D crystal arrays. Imaging them required embedding the protein crystals in another material, such as sugar, that could withstand the vacuum and damaging electron beam inside the microscope. The first demonstration of 2D electron crystallography showed that high-resolution diffraction patterns could be collected from thin protein crystals without the need to stain or fix them using a hydration stage.^2^ That demonstration was followed by the first use of cryo-EM that froze protein crystals and preserved them in a native hydrated state for subsequent electron diffraction studies.^3^

In 1975 Richard Henderson and Nigel Unwin, both at the UK’s Medical Research Council Laboratory of Molecular Biology, presented the first 3D structural models by electron crystallography using glucose-embedded 2D crystals of the purple membrane protein bacteriorhodopsin and bovine-liver catalase at 7 Å and 9 Å resolution, respectively.^4^ They used both imaging and diffraction. Henderson and Unwin extracted phases from Fourier transforms of the images and combined those phases with amplitudes obtained from electron diffraction patterns. Together the phases and amplitudes were then used to reconstruct a 3D density map.^5^ To pull off the achievement, they conducted their experiments with a transmission electron microscope operating at room temperature.

In 1984 Dubochet and collaborators developed a method to rapidly freeze biological specimens by plunging them into liquid ethane.^3^ That procedure freezes the sample and water so quickly that the ice cannot form crystals; it becomes vitrified. The result is a frozen biological specimen that remains in its native hydrated state—an advance in sample-preparation technology that ultimately led to near-atomic-resolution models of bacteriorhodopsin from electron crystallography of cryogenically preserved 2D crystals.^6^ Over the next couple of decades, researchers were able to achieve numerous milestones by using cryo-EM and electron crystallography.

In 2005, biologists resolved the first protein structure—that of aquaporin-0 from “ double-layer” 2D crystals—at near-atomic resolution by using cryo-EM.^7^ To discern the structure of that channel, one of us (Gonen) and collaborators relied on electron crystallography that used only diffraction patterns recorded at various tilt angles. A major advantage of crystallography that can discern single or multiple layers is that membrane proteins can be reconstituted in their native environment. The process allows researchers to study the proteins’ functionality and their interactions in the lipid bilayer.

## Diffraction from tiny 3D crystals

A similar approach revealed the structure of a 3D protein crystal.^8^ The Gonen group collected images of diffraction patterns from crystals of lysozyme at various angles and determined the structure by molecular replacement (see the article by Qun Shen, Quan Hao, and Sol Gruner, Physics Today, March 2006, page 46). The vitrified 3D crystals created small diffraction spots akin to x-ray diffraction experiments.

Gonen and others subsequently modified the approach to record data on a fast camera as the crystal was rotated in the electron beam.^9,10^ Under those circumstances, the procedure was analogous to the standard rotation method in macromolecular x-ray crystallography, which made the data collection better and faster. Continuous rotation in MicroED experiments produced a higher-quality structure of the protein lysozyme from a single microcrystal. And the data could easily be processed using the same software as x-ray crystallography. MicroED data are rapidly collected by continuously rotating vitrified crystals under low-dose conditions in a cryogenically cooled electron microscope.^1^

**FIGURE 2.**
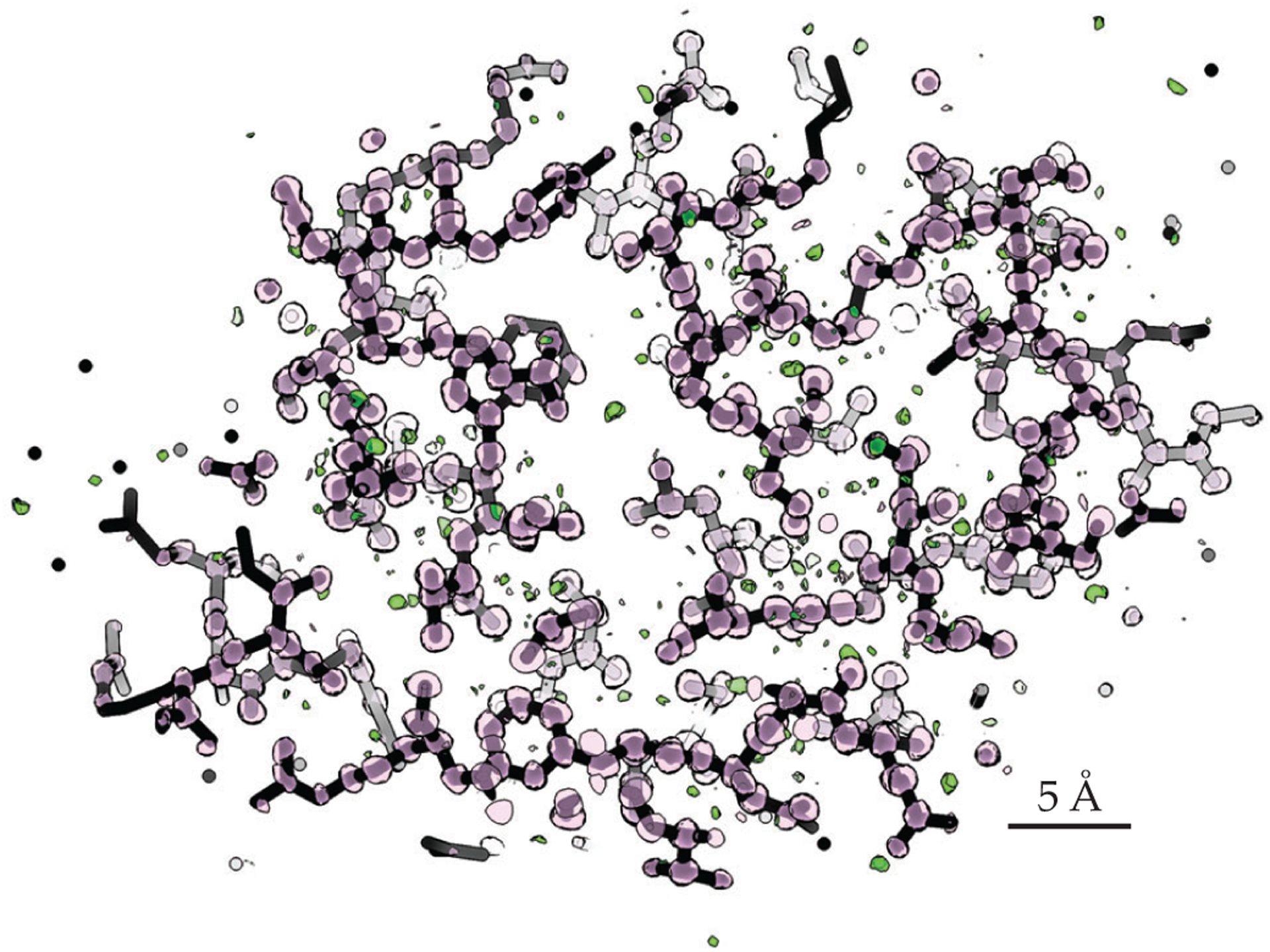
**SUBATOMIC-RESOLUTION STRUCTURE** of triclinic lysozyme. The charge-density map was determined *ab initio*. Pink spheres correspond to protein atoms (carbon, nitrogen, and oxygen, typically), and green spheres correspond to hydrogen atoms. Maps of this quality allow structural biologists to build accurate models of proteins that can aid drug discovery and design. (Adapted from reference 12.)

Following the initial MicroED studies on lysozyme and catalase, which demonstrated the technique’s potential for structural biology, researchers went on to resolve several other structures from 3D protein crystals, including various membrane proteins and ligand-bound complexes.^11^ This past year the two of us and two colleagues demonstrated true atomic resolution from MicroED data on the lysozyme,^12^ shown in figure 2. The demonstration sets the stage for future MicroED studies at subatomic resolution.

Electron crystallography is also a useful technique for resolving the structure of small inorganic and organic molecules. While MicroED researchers adopted the approach and technologies of 2D electron crystallography of proteins, other researchers were using electron diffraction to characterize non-vitrified, radiation-hardy molecules. The two worlds of structural biology and materials science collided in 2018, when two groups independently applied electron diffraction to small-molecule pharmaceutical compounds.^13,14^

In experiments by the two of us and several colleagues,^13^ low-dose conditions were the norm. The conditions facilitated rapid diffraction-data collection and structure determination from beam-sensitive organic molecules. Preparation is relatively straightforward: Samples can be crushed or ground into a dry powder and directly placed on a standard electron microscopy grid for MicroED.

During data acquisition, the grid is exposed to the electron beam, and individual crystals can be selected for MicroED analysis. If the samples being assayed contain mixtures of compounds, the process lets researchers identify the different compounds directly from the mixture at atomic resolution.^13^ That capability opens the field to many possibilities in the study of natural products and the characterization of pharmaceutical compounds.

## What do electrons allow us to see?

Researchers analyzing MicroED data use the same software as those who analyze x-ray experiments. Both methods produce a map, from which an atomic model is built. Although the same software processes the data, the maps generated from the methods provide different information. Whereas x rays scatter from the electron cloud that surrounds an atom, electrons scatter from the atom’s electrostatic potential, which is generated by the interacting positive and negative charges.^15^

**FIGURE 3.**
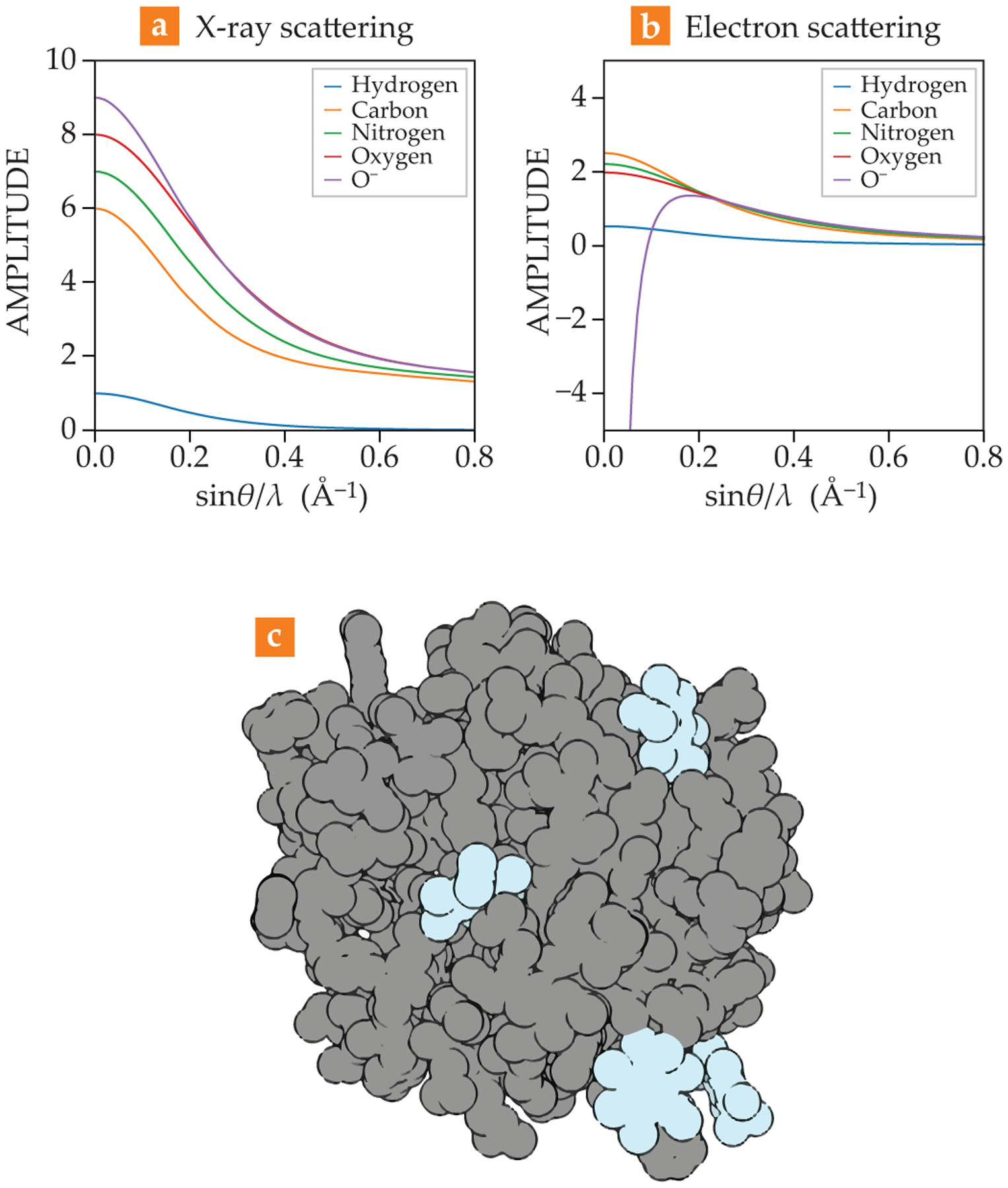
**DIFFERENCES** between **(a)** x-ray and **(b)** electron scattering from neutral and charged atoms. Whereas x rays scatter from an atom’s electron cloud independently, electrons are scattered by the charge environment. Vast differences in scattering can be seen for charged atoms. **(c)** This structure of an enzyme (gray) bound to drugs (blue) was determined by microcrystal electron diffraction.^11^ With those diffraction patterns, researchers can resolve biomolecular structures and screen new drugs and discern how they bind to different proteins.

Because each type of experiment uses different physical phenomena, the information contained in their maps differs. X-ray scattering gives an electron-density map, which reveals where the electrons are inside the crystal. And electron scattering produces a potential map.^16^ That potential depends on both the element and its charge. The local environment can result in wildly different scattering amplitudes from a given atom, as shown in figure 3. Indeed, electron-diffraction experiments can reveal the state of electric charge for amino acids, ions, salts, and even solvent.

The majority of medications approved by the US Food and Drug Administration are molecules with fewer than 70 atoms bound together in a complex 3D shape. Those small-molecule drugs are typically composed of carbon, nitrogen, oxygen, and hydrogen. Hydrogens make up about 50% of the atoms in any given protein or drug. But it’s difficult to resolve the locations of those hydrogens from diffraction patterns taken of proteins and drugs with synchrotron x-ray radiation. That’s because hydrogens are so much lighter than other elements and have a small electron cloud.

Although those atoms can be seen in extremely high-quality data, most structural biology investigations cannot achieve the necessary resolution to accurately find them. Instead, the hydrogen atoms are placed automatically in positions where theoretical considerations suggest they should be located. Scattering using electrons may allow biologists to identify hydrogen atoms at more modest resolutions, because unlike x rays, electrons scatter strongly from hydrogen.

By deciphering where those hydrogens are in a structure,^12,17^ the biologists will be able to model how the drug will bind to the protein receptor of interest. Better binding means that they may design drugs with higher efficacy and fewer side effects. Using MicroED, they can determine the structure of those drugs quickly. Biologists can determine the atomic-resolution structure of the drug bound to the target protein with higher throughput than if they were to attempt to crystallize the drug with the protein beforehand.^18^ The electrostatic-potential map of the bound drug directly reveals how the binding works and how the charges interact. In that respect, MicroED aids the drug-discovery process—by identifying the drug’s structure in order for researchers to understand its interaction with the protein.

## Future of MicroED

**FIGURE 4.**
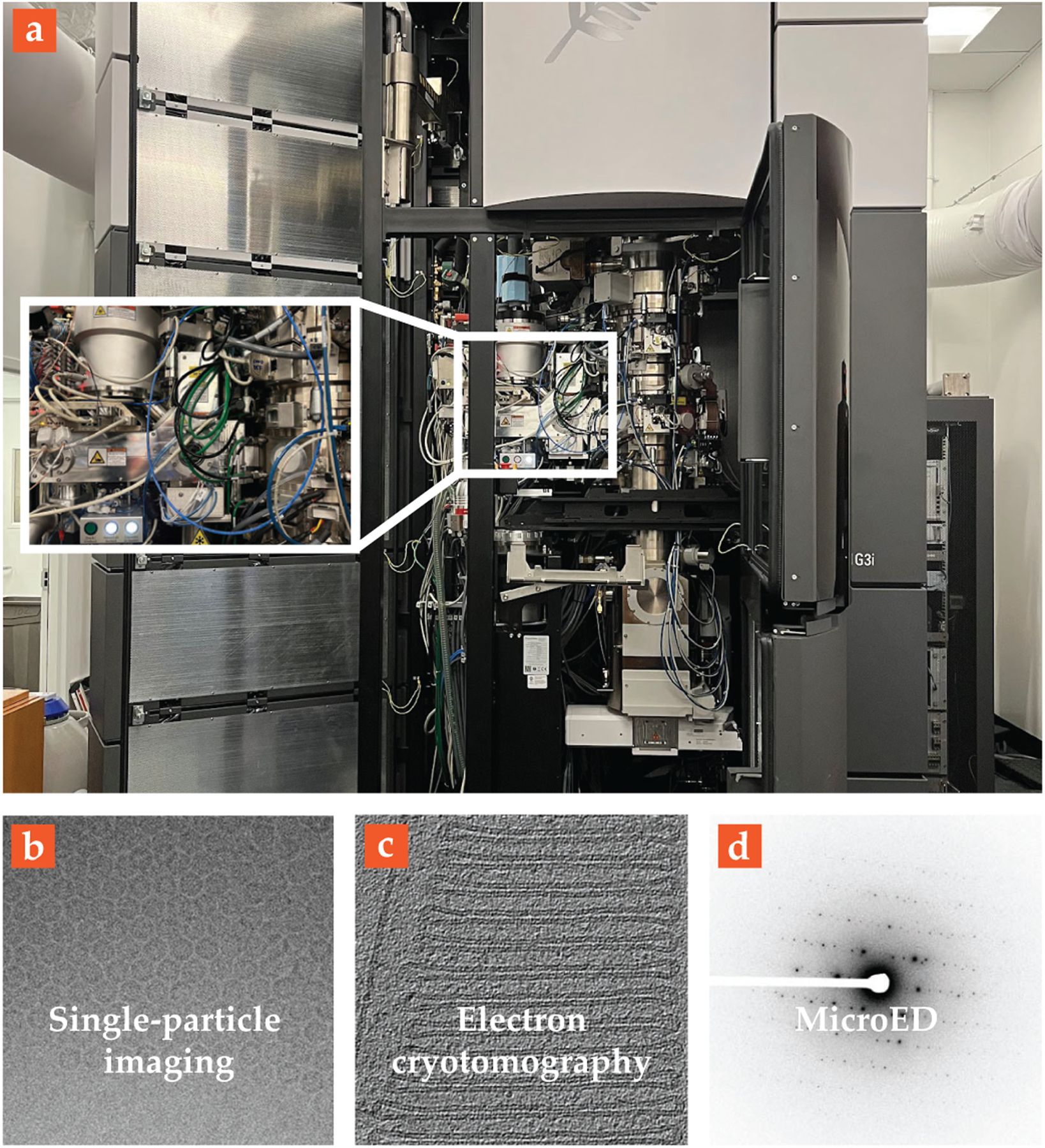
**CRYOGENIC ELECTRON MICROSCOPY**, in practice. **(a)** The internal components of a 300 kV microscope are shown, including (from top to bottom) an electron source, collimated electromagnetic lenses, a cryogenic sample chamber and stage (inset), and several camera systems. The same electron microscope can be used for all modalities of cryogenic electron microscopy. Examples of **(b)** single-particle analysis, **(c)** cryotomography, and **(d)** cryogenic electron diffraction are shown here. In the first two cases, the microscope operates in imaging mode, and a structure is calculated on the basis of the recorded pictures. In the last case, the microscope takes the crystal’s diffraction patterns, from which the structure can be determined. (Panel b adapted from K. M. Yip et al., *Nature* **587**, 157, 2020. Panel c adapted from M. Pöge et al., *eLife* **10**, e72817, 2021.)

The advent of MicroED for proteins and small molecules has created an incredible value for the transmission electron microscope as a structural-biology instrument. The same instrument can be used to take pictures of large proteins and complexes using single-particle and cryogenic electron tomography and to resolve atomic structures from tiny crystals using MicroED, as shown in figure 4. Using just a transmission electron microscope, researchers could feasibly produce an entire drug-discovery pipeline.

The ability to probe charge and visualize potential instead of electron-density maps is not unique to MicroED. It is a property of all electron-microscopy investigations. But reducing a sample to cryogenic temperatures has proven essential for probing the structure of biological materials. Indeed, MicroED opens a new world of structural-biology investigations: Locating hydrogen atoms, accurately modeling electric charge, and determining structures from nanocrystals all give the method an edge in many investigations. The resulting data can inform deep-learning algorithms for solving the protein-folding problem and improve their predictive abilities. (See Physics Today, October 2021, page 14.) Together, such capabilities could lead to rapid improvements in drug discovery. Using the method to determine the structures of molecules that cannot be resolved by any other means is just the beginning.
